# SUPPORT Tools for evidence-informed health Policymaking (STP)

**DOI:** 10.1186/1478-4505-7-S1-I1

**Published:** 2009-12-16

**Authors:** John N Lavis, Andrew D Oxman, Simon Lewin, Atle Fretheim

**Affiliations:** 1 Centre for Health Economics and Policy Analysis, Department of Clinical Epidemiology and Biostatistics, and Department of Political Science, McMaster University, 1200 Main St. West, HSC-2D3, Hamilton, ON, Canada, L8N 3Z5; 2 Norwegian Knowledge Centre for the Health Services, P.O. Box 7004, St. Olavs plass, N-0130 Oslo, Norway; 3 Norwegian Knowledge Centre for the Health Services, P.O. Box 7004, St. Olavs plass, N-0130 Oslo, Norway; Health Systems Research Unit, Medical Research Council of South Africa; 4 Norwegian Knowledge Centre for the Health Services, P.O. Box 7004, St. Olavs plass, N-0130 Oslo, Norway; Section for International Health, Institute of General Practice and Community Medicine, Faculty of Medicine, University of Oslo, Norway

## Abstract

This article is the Introduction to a series written for people responsible for making decisions about health policies and programmes and for those who support these decision makers.

Knowing how to find and use research evidence can help policymakers and those who support them to do their jobs better and more efficiently. Each article in this series presents a proposed tool that can be used by those involved in finding and using research evidence to support evidence-informed health policymaking. The series addresses four broad areas: 1. Supporting evidence-informed policymaking 2. Identifying needs for research evidence in relation to three steps in policymaking processes, namely problem clarification, options framing, and implementation planning 3. Finding and assessing both systematic reviews and other types of evidence to inform these steps, and 4. Going from research evidence to decisions. Each article begins with between one and three typical scenarios relating to the topic. These scenarios are designed to help readers decide on the level of detail relevant to them when applying the tools described. Most articles in this series are structured using a set of questions that guide readers through the proposed tools and show how to undertake activities to support evidence-informed policymaking efficiently and effectively. These activities include, for example, using research evidence to clarify problems, assessing the applicability of the findings of a systematic review about the effects of options selected to address problems, organising and using policy dialogues to support evidence-informed policymaking, and planning policy monitoring and evaluation. In several articles, the set of questions presented offers more general guidance on how to support evidence-informed policymaking. Additional information resources are listed and described in every article. The evaluation of ways to support evidence-informed health policymaking is a developing field and feedback about how to improve the series is welcome.

## About STP

*This article is the Introduction to a series written for people responsible for making decisions about health policies and programmes and for those who support these decision makers. The series is intended to help such people to ensure that their decisions are well-informed by the best available research evidence. The series describes a set of tools that have been developed by the SUPporting POlicy relevant Reviews and Trials (SUPPORT) project, an international collaboration funded by the European Commission’s 6th Framework (http://www.support-collaboration.org). This Introduction describes the SUPPORT tools and the ways in which they can be used. A glossary for the entire series is attached to each article (see additional File *1). *Links to translations of this series into Spanish, Portuguese, French and Chinese can be found on the SUPPORT website (http://www.support-collaboration.org). Feedback about how to improve this series is welcome, and should be sent to: STP@nokc.no. *

## Background

Policymakers and those supporting them often find themselves in situations in which better knowledge about ways to find and use research evidence would help them to do their jobs more effectively and efficiently. In this series, we describe how more systematic processes can be used to support evidence-informed policymaking, identify needs for research evidence, find and assess evidence to address these needs, and go from research evidence to decisions. Here in this introduction to the series, we describe the target audiences for the SUPPORT tools, the proposed tools and how they can be used, what the tools do not do, and how we plan to support their further development.

## The target audiences for the SUPPORT tools

The SUPPORT tools presented in this series have been developed primarily for policymakers and those who support them.

Policymakers are a diverse group that includes cabinet members (e.g. Ministers of Health or Finance), elected officials (e.g. chairs of legislative committees), senior civil servants (e.g. directors of primary healthcare programmes), and high-level political appointees (e.g. heads of government agencies). Policymakers may differ significantly on the basis of their authority or role in different political systems but what all have in common is the authority to make or influence decisions directly. In some countries, cabinet members may be elected, whereas the senior civil servants who advise them may be neutral advisors with no affiliations to the governing party. In other countries, all positions carrying decision-making authority may be appointed by the governing party. Policymakers may also differ by sector (e.g. health or economy) or operational level (e.g. local or national).

Those who support policymakers are equally diverse and may include individuals within government (e.g. junior civil servants such as policy analysts, or the political staff of an elected official or high-level political appointee), and individuals working in independent units that provide support for the use of research evidence in policymaking. But their role in informing the decisions made by policymakers is common to all. This, despite the fact that they may differ is in their degree of independence from policymakers (e.g. a semi-autonomous government agency, or a health systems research unit that is independent of government but supports the use of research evidence in policymaking) and their affiliation with other institutions (e.g. non-governmental organisations, universities).

The SUPPORT tools are also relevant to health system stakeholders. This group may include non-governmental organisations and civil society groups that play a diverse range of roles. They may, for example, seek to influence decisions made by policymakers. Or else they work in areas not normally addressed by policymakers, or in areas where authority has been delegated to them by policymakers themselves. We recognise, though, that some of the language and examples used in this series may resonate more with policymakers and those who support them.

The SUPPORT tools have been written for settings that range from low- and middle-income countries such as Uganda and Chile, to high-income countries such as Canada and Norway. Wherever possible, examples have been drawn from disparate settings. As described below, many of the issues and opportunities encountered in supporting evidence-informed policymaking are remarkably similar across settings.

Each article begins with between one and three typical scenarios designed to encourage readers to use the tools described and to help them to decide on the relevant level of detail they require. Some scenarios describe senior civil servants who simply need a general sense of the expectations required for their staff – this information can be quickly gained by them by scanning through the article. Other scenarios, for example, relate to junior policy analysts and directors of an applied health systems research units. They will require more specific guidance on how to undertake new activities and should therefore read the relevant article in more detail when asked to undertake such tasks. The article will also be useful to them as a reference.

## The SUPPORT tools and how they can be used

**Figure 1:**
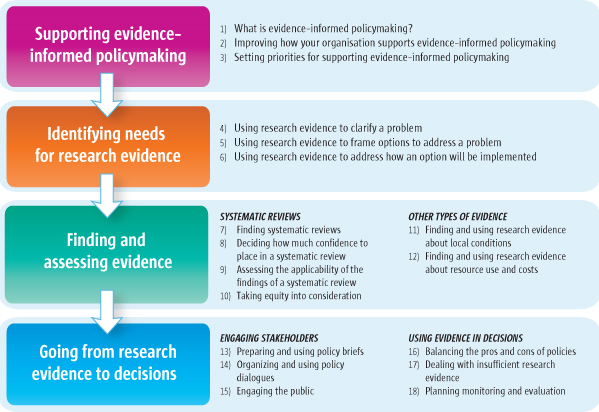
Overview of the series

In each article in this series, we propose a tool that can be used by those involved in finding and using research evidence to support evidence-informed health policymaking. The series addresses four broad areas of interest related to policymaking: 1. Supporting evidence-informed policymaking (Articles 1-3 [1-3]) 2. Identifying needs for research evidence in relation to three steps in policymaking processes, namely clarifying problems, framing options and planning implementation (Articles 4-6 [4-6]) 3. Finding and assessing evidence to inform each of these steps (Articles 7-10 [7-10] focus on systematic reviews and Articles 11-12 [11,12] on other types of evidence) 4. Going from research evidence to decisions (Articles 13-15 [13-15] focus on engaging stakeholders in evidence-informed policymaking. Articles 16-18 [16-18] address how to use research evidence in decisions). Figure 1 provides an overview of the series, with the numbers shown referring to the relevant article. Additional resources and website links are provided in each article.

In the articles on supporting evidence-informed policymaking (the first of the four key areas covered in this series), the principal focus of each is a set of questions that can be used to guide ways to support evidence-informed policymaking. Policymakers and those who support them may wish to know more about what evidence-informed policymaking is (Article 1) [1], how to improve the ways that their organisation supports evidence-informed policymaking (Article 2) [2] or how to set priorities for supporting evidence-informed policymaking (Article 3) [3]. These articles can help to guide those striving to understand and to shape the context for evidence-informed policymaking.

In the other three broad areas (see Figure 1), the main focus of each article is a set of questions that can guide how an activity that supports evidence-informed policymaking can be undertaken. Such activities might include using research evidence to clarify a problem (Article 4) [4], assessing the applicability of the findings of a systematic review about the effects of a policy or programme option to address a problem (Article 9) [9], organising and using policy dialogues to support evidence-informed policymaking (Article 14) [14], and planning the monitoring and evaluation of policies (Article 18) [18].

Those who wish to learn about the different types of research evidence needed in policymaking processes should ideally start with Articles 4-6 [4-6]. These three articles correspond to three steps policymaking processes, namely problem clarification, option framing and implementation planning. These articles in the series help to identify needs for research evidence in relation to each of these steps. While policymaking processes rarely involve a clear sequence of steps, even highly dynamic processes can benefit from a systematic method of clarifying a problem, framing options to address it, and defining how an option will be implemented. Article 13 [13] – which we return to below – describes how to bring these steps together in policy briefs which are used to support the use of research evidence in policymaking.

Those familiar with how to identify needs for research evidence in relation to each step in a policymaking process, as well as those needing to undertake a more focused activity related to finding and assessing evidence, may want to move directly to one or more of Articles 7-12 [7-12]. Within these articles, there are two sub-series of articles. The first addresses the following issues related to systematic reviews:

• How to find systematic reviews (Article 7) [7]. Policymakers and those who support them will need to understand the rationale for seeing systematic reviews as a ‘first place to look’ and how to find them efficiently

• How much confidence can be placed in a systematic review (Article 8) [8]. Like any type of research, a systematic review can be conducted and reported well or poorly. Policymakers will want to know the reliability of a review that supports an option that they will be endorsing

• How to assess the applicability of the findings of a systematic review (Article 9) [9]. Those who support policymakers will need to assess whether the findings of a review of studies conducted in very different settings, do actually apply in their own setting

• How to take equity into consideration when assessing the findings of a systematic review (Article 10) [10]. Many policymakers will wish to consider the potential impacts of an option on disadvantaged groups or settings.

Article 7 [7] emphasises the merits of systematic reviews, while the three articles that follow, grapple with the challenges of using reviews in policymaking. Articles 11 and 12 together complete a second sub-series about finding and using research evidence about local conditions (Article 11) [11] and resource use and costs (Article 12) [12].

Once research evidence has been found and assessed, a variety of opportunities and issues may arise when going from research evidence to a decision. This issue is the focus of Articles 13-18 [13-18]. These articles contain two additional sub-series of three articles each. The first examines ways to engage stakeholders to support evidence-informed policymaking. Two introduce new innovations:

• Policy briefs that package research evidence so as to inform deliberations among policymakers and stakeholders (Article 13) [13]

• Policy dialogues that allow research evidence to be considered together with the views, experiences and tacit knowledge of those who will be involved in, or affected by, future decisions about a high-priority issue (Article 14) [14]

Research evidence is only one factor that can influence the policymaking process. Policy dialogues provide an opportunity to discuss research evidence as well as the many other factors that can exert influence. The third article focuses on how to engage the public in evidence-informed policymaking (Article 15) [15].

The second and final sub-series addresses issues related to using research evidence in decisions. These are:

• Using research evidence in balancing the pros and cons of policies (Article 16) [16]

• Dealing with insufficient research evidence (Article 17) [17], and

• Planning the monitoring and evaluation of policies (Article 18) [18]

The last article in this series could also be read in conjunction with the articles about problem clarification (Article 4) [4], options framing (Article 5) [5] and implementation planning (Article 6) [6]. Planning monitoring and evaluation is arguably a fourth step in policymaking processes.

Some issues, such as equity, are a recurring theme in many of the articles even if they are the primary focus of only one article (Article 10) [10].

## What the SUPPORT tools do not do

The SUPPORT tools have been developed giving due consideration to other features of the policymaking process. For example, the article about using research evidence to clarify a problem (Article 4) [4] notes the importance of watching for windows of opportunity that may arise due to political events, such as a shifts or changes in the balance of organised political forces or the appointment of a new health minister. Article 9 [9] examines how to assess the applicability of the findings of a systematic review and notes the importance of evaluating whether the studies included in a systematic review were conducted in settings with largely similar perspectives and political influence amongst health system stakeholders, compared to the settings to which policy decisions may be applied.

The SUPPORT tools do not, however, address efforts to support health policymaking in general. As the titles indicate, the focus of each tool is on supporting *the use of research evidence in* health policymaking. This does not mean that other forms of support could not complement these tools. Policymakers, for example, also need to know how to assess and influence stakeholder dynamics (independent of the implications of such dynamics for the applicability of the findings of a systematic review). Such dynamics, including power relations among stakeholders and the interests of these different groups, are a key factor influencing the policymaking process. Values are another domain where tools to support their systematic and explicit consideration in health policymaking could be useful for policymakers and those who support them.

By focusing on how to support the use of research evidence in health policymaking, the SUPPORT tools are meant to aid the use of the best research evidence available at the time that it is needed and in the time available to compile such evidence. Research evidence may be lacking, incomplete, imperfect and even contradictory. But policymakers still need to make decisions. Proceeding on the basis of available research evidence, with an awareness of its strengths and limitations, would be seen by many stakeholders as an indication that the work of policymakers was appropriate and constructive. Monitoring how options are implemented, evaluating their impacts, and later making adjustments as better research evidence becomes available, would further this impression.

## Further development of the SUPPORT tools

Some of the activities and broader efforts to support evidence-informed policymaking that are addressed in the SUPPORT tools have received considerable attention in research. For example, the SUPPORT tool that is used for finding and using research evidence about resource use and costs (Article 12) [12] draws on relevant aspects of the economic evaluation literature. Further, some of the SUPPORT tools have already been used extensively in the field and adapted iteratively based on these experiences. For example, successive iterations of the SUPPORT tool for using research evidence to frame options to address a problem (Article 5) [5] have been used in workshops for policymakers, stakeholders and researchers from more than ten countries in Africa, four countries in Asia, and seven countries in the Americas. For us, this field testing has reinforced the fact that many of the issues and opportunities encountered in supporting evidence-informed policymaking are remarkably similar across settings.

Other activities and support efforts have received less attention. For example, SUPPORT tools such as those that address how to improve the ways that an organisation supports evidence-informed policymaking (Article 2) [2], how an organisation sets priorities for supporting evidence-informed policymaking (Article 3) [3], how to prepare and use policy briefs (Article 13) [13], and how to organise and use policy dialogues (Article 14) [14] would all benefit from the development of a more rigorous evidence base. These tools have also been subjected to less field-testing thus far. We are disseminating the full set of tools in anticipation that wider use and application will inform further adaptation. Feedback on how to improve the tools is welcome. We would also welcome feedback about what tools need to be added to the series.

## Conclusion

The SUPPORT tools in this series have been designed to help policymakers and those who support them to do one aspect of their job better or more efficiently – namely to find and use research evidence to support health policymaking. The tools are also relevant to health system stakeholders, such as non-governmental organisations and civil society groups. Different readers will use the tools in different ways. Policymakers may skim the articles to get ideas on how they should be adjusting the expectations they set for their staff. Those who support policymakers may choose to read a particular article to help them with undertaking a new activity, and then use the article later as a reference guide or as a way of refining their skills. We hope that policymakers and those who support them will help us to develop and improve what is presented here.

## Resources

### Useful documents and further reading

- Lavis JN, Oxman AD, Moynihan R, Paulsen E. Evidence-informed health policy:1. Synthesis of findings from a multi-method study of organizations that support the use of research evidence. Implementation Science 2008, 3:53: http://www.implementationscience.com/content/3/1/53 – Source of insights from organisations actively engaged in supporting the use of research evidence in policymaking, particularly policymaking in low- and middle-income countries

- Sutcliffe S, Court J. A Toolkit for Progressive Policymakers in Developing Countries. London, UK: Overseas Development Institute, 2006: http://www.odi.org.uk/resources/download/154.pdf – Source of additional tools that can be used by those supporting policymaking, particularly policymaking in low- and middle-income countries

- Ciliska D, Thomas H, Buffett C. Introduction to Evidence-Informed Public Health and a Compendium of Critical Appraisal Tools for Public Health Practice. Hamilton, Canada: National Collaborating Centre for Methods and Tools, 2008: http://www.nccmt.ca/pubs/2008_07_IntroEIPH_compendiumENG.pdf - Source of additional tools that can be used by those supporting the use of research evidence in policymaking, particularly related to public health

- Guyatt G, Rennie D, Meade MO, Cook DJ (Editors). Users’ Guides to the Medical Literature: A Manual for Evidence-Based Clinical Practice. Second Edition. New York, USA: McGraw Hill Medical, 2008 – Source of additional tools that can be used by those supporting the use of research evidence in policymaking, particularly in policymaking related to clinical care

- Research Matters. Knowledge Translation: A ‘Research Matters’ Toolkit. Ottawa, Canada: International Development Research Centre: http://www.idrc.ca/research-matters/ev-128908-201-1-DO_TOPIC.html – Source of additional tools that can be used by those supporting the use of research evidence in policymaking, particularly by researchers

### Links to websites

- SUPporting POlicy relevant Reviews and Trials (SUPPORT) Collaboration: http://www.support-collaboration.org – Source of translations of this series into Spanish, Portuguese, French and Chinese.

## Competing interests

The authors declare that they have no competing interests.

## Authors’ contributions

JNL prepared the first draft of this article. ADO, SL and AF contributed to drafting and revising it.

## Supplementary Material

Additional file 1 Glossary Click here for file
