# Supplementary material for: SUPPORT Tools for evidence-informed health Policymaking (STP)
Source: Health Res Policy Syst. 2009 Dec 16;7(Suppl 1):I1. doi: 10.1186/1478-4505-7-S1-I1 (PMC3271819; doi:10.1186/1478-4505-7-S1-I1)
Supplement: Additional file 1 — Glossary [file 1478-4505-7-S1-I1-S1.doc]

**Glossary**

For additional definitions see the list of further glossaries at the end of this glossary.

**Absolute effectiveness**: (See Table 4, STP 10) also absolute effect

**Acceptability (related to indicators)**: The extent to which the indicator is acceptable to those being assessed and those undertaking the assessment

**AMSTAR (A MeaSurement Tool to Assess Reviews)**: A tool designed to assess the quality of the methods used to conduct a systematic review (see [www.biomedcentral.com/1471-2288/7/10](http://www.biomedcentral.com/1471-2288/7/10))

**ART**: antiretroviral therapy. Also referred to as HAART – highly active antiretroviral therapy

**Balance sheet**: See discussion in Table 1, STP 16

**Baseline conditions (also known as baseline characteristics)**: Values of demographic, clinical and other variables collected for each participant at the beginning of a trial, before the intervention is administered

**Baseline risk**: The risk (likelihood) of an outcome without implementing an intervention or at the beginning of a study

**Bayesian meta-analysis**: An approach to statistics based on application of Bayes’ theorem that can be used in single studies or meta-analysis. A Bayesian analysis uses Bayes' theorem to transform a prior distribution for an unknown quantity (e.g. an odds ratio) into a posterior distribution for the same quantity, in light of the results of a study or studies. The prior distribution may be based on external evidence, common sense or subjective opinion. Statistical inferences are made by extracting information from the posterior distribution, and may be presented as point estimates, and credible intervals (the Bayesian equivalent of confidence intervals)

**Burden of disease**: This refers to the impacts (or burden) of a health problem or condition (such as hypertension) in an area (such as a country or province), as measured by mortality, morbidity or other indicators. Burden of disease is sometimes measured using the ‘disability-adjusted life year’ or DALY – a time-based measure combining years of life lost due to premature mortality and years of life lost due to time lived in states of less than full health

**Case survey**: A method for synthesising findings from a number of qualitative studies or to combine qualitative and quantitative evidence within a single review. It involves the systematic coding of relevant data from the included qualitative case studies and the subsequent conversion of these codes into a quantitative form. This then allow statistical analysis

**CASP (Critical Appraisal Skills Programme)**: A UK-based programme that aims to enable individuals to develop the skills to find and make sense of research evidence, thereby helping them to put knowledge into practice (see: [www.phru.nhs.uk/pages/PHD/CASP.htm](http://www.phru.nhs.uk/pages/PHD/CASP.htm))

**Chi-squared test for homogeneity**: A statistical test based on comparison of a test statistic to a chi-squared distribution used in meta-analyses to test the statistical significance of **heterogeneity** (see ‘Heterogeneity’)

**CHSRF**: Canadian Health Services Research Foundation

**Confidence interval (CI)**: A confidence interval is a range around an estimate that conveys how precise the estimate is; for example an estimate of the risk of an event occurring or an estimate such as a risk ratio that compares the risk with and without an intervention. The confidence interval is a guide to how sure we can be about the quantity we are interested in. The narrower the range between the two numbers, the more confident we can be about what the true value is; the wider the range, the less sure we can be. The width of the confidence interval reflects the extent to which chance may be responsible for the observed estimate (with a wider interval reflecting more chance)

**Control group**: A group of participants in a study not receiving a particular intervention, used as a comparator to evaluate the effects of the intervention (see ‘Intervention group’)

**Controlled before-after study**: A **non-randomised study** design where a control **population** of similar characteristics and performance as the **intervention group** is identified. Data are collected before and after the **intervention** in both the **control** and intervention groups

**Controlled trial** (See trial)

**Cost-effectiveness analysis**: An economic evaluation in which the costs and consequences of alternative interventions are expressed cost per unit of health outcome (e.g. cost per additional stroke prevented) (for a more detailed discussion see Table 2, STP 5)

**Cross-case techniques**: A method for synthesising findings across a number of qualitative studies or cases

**Deflator**: A statistical factor designed to remove the effect of inflation

**Differential effectiveness**: Different degrees of effectiveness (or adverse effects) in different groups or settings

**Disaggregated data**: Often data are reported for whole populations or areas. This can be referred to as aggregate data. In some cases, it may be desirable and possible to further breakdown or analyse these data to look at specific groups (such as people over the age of 65 years) or areas (such as a health district). This can be referred to as disaggregated data

**Effectiveness**: The extent to which a specific intervention, when used under ordinary circumstances, does what it is intended to do

**Egger regression test**: A statistical method for detecting publication bias in a review or meta-analysis. The test has a similar purpose to that of the funnel plot (see ‘funnel plot’)

**Empirical evidence**: Empirical results based on observation rather than on reasoning alone

**Epidemiology**: The study of the health of populations and communities, not just particular individuals

**Equity considerations**: Attention to how a policy or programme may impact on inequities (see inequity) (for a more detailed discussion see STP 10)

**Estimate of effect**: The observed relationship between an intervention and an outcome expressed as, for example, a number needed to treat to benefit, odds ratio, risk difference, risk ratio, standardised mean difference, or weighted mean difference

**Evaluation**: a term often used interchangeably with monitoring. The former usually suggests a stronger focus on the achievement of results

**Evidence-based medicine (EBM)**: Evidence-based medicine is the conscientious use of current best evidence in making decisions about the care of individual patients or the delivery of health services. The terms ‘evidence-based health care’ and ‘evidence-based practice’ are often used interchangeably with ‘evidence-based medicine’ (see STP 1)

**Evidence-informed health policymaking**: Evidence-informed health policymaking is an approach to policy decisions that aims to ensure that decision making is well-informed by the best available research evidence. It is characterised by the systematic and transparent access to, and appraisal of, evidence as an input into the policymaking process (see STP 1)

**Feasibility (related to indicators)**: The extent to which valid, reliable and consistent data are available for collection

**Funnel plot**: A graphical display of some measure of study precision plotted against effect size that can be used to investigate whether there is a link between study size and treatment effect. One possible cause of an observed association is reporting bias. The plot is therefore often used to assess whether publication bias is likely within a systematic review

**GRADE assessment system**: See Table 8, STP 16

**Grey literature**: Grey literature is the kind of material that is not published in easily accessible journals or databases. It includes things like conference proceedings that include the abstracts of the research presented at conferences, unpublished theses, and so on

**Grounded theory**: An approach used widely within primary qualitative research and focused on the generation of theory or explanations for social phenomena, based on empirical data. The method can also be used to synthesise findings across a number of qualitative studies

**Health status**: The state of health of a person or population assessed with reference to morbidity, impairments, anthropological measurements, mortality, and indicators of functional status and quality of life

**Health system arrangements**: The delivery, financial and governance arrangements within which clinical or public health programmes and services are provided

**Health technology assessment (HTA)**: HTA is the systematic evaluation of the properties, effects and/or other impacts of health care technology. Its primary purpose is to provide objective information to support healthcare decisions and policymaking at the local, regional, national and international levels. HTA reports typically include a range of economic, social, ethical and legal considerations, as well as a review of the research evidence about the effectiveness of a technology. Some HTA reports contain a systematic review that can be applied in contexts other than the one for which the report was produced

**Hedges**: Validated search strategies to find specific types of single studies

**Heterogeneity**: 1. Used in a general sense to describe the variation in, or diversity of, **participants**, **interventions**, and measurement of outcomes across a set of studies, or the variation in internal **validity** of those studies. 2. Used specifically, as statistical heterogeneity, to describe the degree of variation in the **effect estimates** from a set of studies. Also used to indicate the presence of variability among studies beyond the amount expected due solely to the play of chance

**Impact evaluation**: An evaluation that aims to determine whether the observed changes in outcomes (or “impact”) can be attributed to a particular policy or programme

**Indicator**: A quantitative or qualitative factor or variable that provides a simple and reliable means to measure achievement, to reflect the changes connected to an intervention, or to help assess the performance

**Indirect evidence**: Research that has not directly compared the options in which we are interested in the populations in which we are interested, or measured the important outcomes in which we are interested

**Inequity in health**: A difference in health that is not only unnecessary and avoidable but, in addition, is considered unfair and unjust

**Intermediary outcome**: Outcome measures that are not of direct practical importance but are believed to reflect outcomes that are important; for example, blood pressure is not directly important to patients but it is often used as an outcome in clinical trials because it is a risk factor for stroke and heart attacks. Surrogate endpoints are often physiological or biochemical markers that can be relatively quickly and easily measured, and that are taken as being predictive of important outcomes. They are often used when observation of important outcomes requires long follow-up

**Interrupted time series analysis or study**: A research design that collects observations at multiple time points before and after an intervention (interruption). The design attempts to detect whether the intervention has had an effect significantly greater than the underlying trend

**Intervention**: The process of intervening on people (e.g. clinical interventions), groups or entities (e.g. health policy or programme options)

**Intervention group**: A group of participants in a study receiving a particular policy or programme option

**LMIC**: Low- or middle-income country

**MESH term (or headings)**: An abbreviation for Medical Subject Headings) Terms used by the United States National Library of Medicine to index articles in MEDLINE. The MeSH system has a tree structure in which broad subject terms branch into a series of progressively narrower subject terms

**Meta-analysis**: The use of statistical techniques in a **systematic review** to integrate the results of included studies. Sometimes used as a synonym for systematic reviews, where the review includes a meta-analysis

**Meta‑ethnography**: A method of translating ideas, concepts and metaphors across different qualitative studies in order to synthesise their findings. The method draws on the ethnographic approach used in primary qualitative research

**Modifying factor**: A factor or characteristic, such as the size of a health facility that may change or modify the effect of the proposed causal factor being studied, such as health worker motivation

**Monitoring/performance monitoring**: describes the process of systematically collecting data to inform policymakers, managers and other stakeholders whether a new policy or programme is being implemented in accordance with their expectations

**Narrative review**: A summary in words (rather than numerically) of, for example, the effects of a policy or programme option. Narrative reviews are not always based on a thorough and reproducible search of the literature for studies that address the review question

**Narrative summary/synthesis**: See ‘Narrative review’. The approach can be used to synthesise findings across a number of qualitative studies or to combine qualitative and quantitative evidence within a single review

**Observational study**: A study in which the investigators do not seek to intervene, and simply observe the course of events. Changes or differences in one characteristic (e.g. whether or not people received the intervention of interest) are studied in relation to changes or differences in other characteristic(s) (e.g. whether or not they died), without action by the investigator. There is a greater risk of selection bias than in experimental studies. See also randomised controlled trial. (Also called non-experimental study)

**OECD**: Organisation for Economic Co-operation and Development

**Outcome**: A change resulting from an intervention. In evaluations, a potential consequence of an intervention that is measured after the intervention has been implemented, that is used to assess the effects of the intervention

**PICO (Population, Intervention, Comparison, Outcomes)**: The acronym, PICO, is used to summarise the four key components of a review or research question. In this series we also introduce the acronym, POCO, replacing intervention with option. POCO similarly summarises the four key components of a question

**Policy brief**: See discussion in STP 13

**Policy dialogue:** (see discussion in STP 14)

**Policy entrepreneur**: People who seek to initiate policy change

**Primary outcome**: The outcome of greatest importance

**Primary study**: ‘Original research’ in which data are collected. The term primary study is sometimes used to distinguish it from a secondary study (re-analysis of previously collected data), meta-analysis, and other ways of combining studies (such as economic analysis and decision analysis). (Also called original study)

**Process evaluation**: Process evaluations explore the delivery of a process or programme and the mechanisms underlying its effects. They verify what the policy or programme is and whether or not it is delivered as intended to the target recipients

**Process indicator**: An indicator for actions taken or work performed through which inputs, such as funds, technical assistance and other types of resources are mobilised to produce specific outputs (see ‘Indicator’). Processes may also be referred to as ‘activities’ in the results chain (see Figure 1, STP 18)

**Publication bias**: A bias caused by only a subset of all the relevant data being available. The publication of research can depend on the nature and direction of the study results. Studies in which an intervention is not found to be effective are sometimes not published. Because of this, systematic reviews that fail to include unpublished studies may overestimate the true effect of an intervention. In addition, a published report might present a biased set of results (e.g. only outcomes or sub-groups where a statistically significant difference was found

**Purchasing power parity (PPP)**: A criterion for an appropriate exchange rate between currencies

**Qualitative comparative analysis**: A method for synthesising findings from a number of qualitative studies or to combine qualitative and quantitative evidence within a single review

**Qualitative study**: Qualitative approaches attempt to describe and interpret human phenomena rather than to measure these. These methods focus on finding answers to questions centred on social experience, including the values and perceptions of individuals and groups and how they experience the world around them, including health care

**Randomised controlled trial (RCT)**: An experiment in which two or more interventions, possibly including a control intervention or no intervention, are compared by being randomly allocated to participants

**Randomised trial**: (see ‘randomised controlled trial’)

**RCT**: Randomised controlled trial

**Realist review/synthesis**: A theory-based method for synthesising findings from a number of qualitative studies

**Relative effectiveness** (See Table 4, STP 10)

**Relative reduction**: See relative effectiveness

**Results chain**: The causal sequence for a development intervention that stipulates the necessary sequence to achieve desired objectives – beginning with inputs, moving through activities and outputs, and culminating in outcomes, impacts, and feedback. In some agencies, reach is part of the results chain

**Retrospective analysis/study**: An analysis or study planned and conducted after the dataset has already been collected. For example, routinely collected data may be analysed retrospectively to evaluate the effects of a new programme

**Risk factor**: An aspect of a person's condition, lifestyle or environment that affects the **probability** of occurrence of a disease. For example, cigarette smoking is a risk factor for lung cancer

**Routine data**: Data or information collected as part of normal health service management, monitoring and evaluation. This may include information on the prevalence of diseases, on healthcare utilisation, or on service costs

**Stakeholder**: A person, group or organisation that has a legitimate interest in or can be affected by a health policy or programme

**Statistical pooling**: The use of quantitative, statistical methods to combine the findings of a number of studies of the effects of programme or policy options. This is also referred to as meta-analysis and may be part of a systematic review

**Statistical significance**: The likelihood that a finding or a result is caused by something other than just chance (see Table 2, STP 17)

**Subgroup analysis**: An analysis in which the intervention effect is evaluated in a defined subset of the participants in a study or systematic review, or in complementary subsets, such as by sex or in age categories

**Surrogate outcome**: Outcome measures that are not of direct practical importance but are believed to reflect outcomes that are important; for example, blood pressure is not directly important to patients but it is often used as an outcome in clinical trials because it is a risk factor for stroke and heart attacks. Surrogate endpoints are often physiological or biochemical markers that can be relatively quickly and easily measured, and that are taken as being predictive of important outcomes. They are often used when observation of important outcomes requires long follow-up

**Systematic review**: Summaries of research evidence that address a clearly formulated question using systematic and explicit methods to identify, select, and critically appraise relevant research, and to collect and analyse data from the studies that are included in the review

**Thematic analysis**: A method for synthesising findings from a number of qualitative studies. It includes identifying key themes in the included studies and then summarising the evidence within these themes or categories

**Trial**: The term ‘trial’ is sometimes used to refer to randomised controlled trials (RCTs). The term may also be used to refer to quasi-randomised trials (e.g. where alternation (every other person or group) is used to allocate people, rather than randomisation) (Also called controlled trial)

**Validity (related to indicators)**: The extent to which an indicator accurately measures what it purports to measure

**Further glossaries relevant to evidence-informed health policymaking**

Canadian Health Services Research Foundation Glossary of knowledge exchange terms as used by the Foundation: [www.chsrf.ca/keys/glossary_e.php](http://www.chsrf.ca/keys/glossary_e.php)

Canadian Institutes of Health Research KT Clearinghouse Glossary: <http://ktclearinghouse.ca/glossary>

Cochrane Collaboration Glossary of Cochrane Collaboration and research terms: [www.cochrane.org/resources/glossary.htm](http://www.cochrane.org/resources/glossary.htm)

European Observatory on Health Systems and Policies Glossary: [www.euro.who.int/observatory/glossary/toppage](http://www.euro.who.int/observatory/glossary/toppage)

Global Forum for Health Research Glossary: [www.globalforumhealth.org/Glossary](http://www.globalforumhealth.org/Glossary)

Health Economics Information Resources Glossary of frequently encountered terms in health economics: <http://www.nlm.nih.gov/nichsr/edu/healthecon/glossary.html>

National Institute of Health Policy Glossary of health policy terms: [www.nihp.org/NEWglossary.htm](http://www.nihp.org/NEWglossary.htm)

NHS Evidence Glossary of health knowledge management terms: [www.library.nhs.uk/knowledgemanagement/page.aspx?pagename=GLOSSARY](http://www.library.nhs.uk/knowledgemanagement/page.aspx?pagename=GLOSSARY)

Public Health Agency of Canada: <http://cbpp-pcpe.phac-aspc.gc.ca/glossary/all_terms-eng.html>

WhatisKT: <http://whatiskt.wikispaces.com/>
